# Application of multimodal identification technology in the innovative management operation department

**DOI:** 10.3389/fsurg.2022.964985

**Published:** 2022-09-23

**Authors:** Yan Zhu, Xiaojiao Sun, Yuemei Huang, Xiaochong Song, Li Liu, Laide Feng, Yujian Zhang

**Affiliations:** ^1^ Operating Room, the First Hospital of Qinhuangdao, Qinhuangdao, China; ^2^ Nose and Throat Department, the First Hospital of Qinhuangdao, Qinhuangdao, China; ^3^ Head of the Surgical Department, the First Hospital of Qinhuangdao, Qinhuangdao, China

**Keywords:** laparoscopic surgery, operation department, multimodal identification technology, operating efficiency, frequency

## Abstract

**Background:**

The optimization of surgical procedures and the management of surgical quality and safety have become the focus of attention of hospital managers. The application of multimodal identification technology in the innovative management mode of hospital operating department has made remarkable progress.

**Methods:**

To investigate the effect of the upgraded multimodal identification technology on the innovative management of the operating department, 2,280 cases of laparoscopic surgery using traditional surgical management procedures from January to December 2019 before the management upgrade were set as the control group, and 2,350 laparoscopic surgeries with the upgraded multimodal identification management process from January to December 2020 were selected as the experimental group. The operating efficiency, material management efficiency, and patient experience and satisfaction of the two groups were investigated and compared.

**Results:**

Compared with traditional procedures, the upgraded multimodal surgical management system significantly improves the efficiency of laparoscopic surgery and reduces surgical consumption and costs. In addition, the multimodal surgical information identification system significantly improves the surgical experience for patients undergoing laparoscopic surgery.

**Conclusion:**

Application of multimodal identification technology improves the innovative management of operation department compared with traditional surgery management procedure.

## Introduction

Operating room management is a complex subject (1). The optimization of surgical procedures and the management of surgical quality and safety have become the focus of attention of hospital managers around the world (2, 3). As a public service platform for hospital surgery, the operating department is the core of the surgical management process (4). The management process of the operating department, the behavior of surgical staff, the quality of material supply, and the operational efficiency all affect the quality and safety of surgical patients and their medical experience to varying degrees (5, 6).

The multi-modal surgical information identification system builds an intelligent management platform for the operating department (7). The surgical department of our hospital has upgraded the traditional operating room management system to a multi-modal surgical information identification system since 2020. Our multi-modal surgical information identification system establishes a data connection with the hospital management information system, realizes real-time information update and ensures that all patient information is true and accurate (8). The multi-modal surgical information identification system can also reduce manual operations as much as possible through operations such as staff card swiping, mobile application scanning code identification, and system-side appointments, so as to minimize human interference factors and ensure the standardization, efficiency and safety of the entire surgical process information.

With the rapid development of medical technology, laparoscopic surgery has been widely used in the treatment of general surgery patients (9, 10). Laparoscopic surgery has the advantages of small incision, less blood loss and quick recovery (11, 12). However, various complications of laparoscopic surgery, such as postoperative infection, bleeding, nausea and vomiting, and shock, will affect the patient's physical recovery (13). Therefore, formulating an effective procedure and nursing management process is a necessary means to reduce the waiting time of patients, improve the efficiency of laparoscopic surgery, and accelerate the recovery of patients (14, 15).

In this study, we hypothesized that our upgraded multi-model information management technology can effectively prompt laparoscopic surgery efficiency and reduce consumables consumption compared with our previous traditional surgical management. Our research aims to solve the problems in actual hospital management by comparing the traditional operation turnover process and the innovative management mode of multi-information identification technology, and improve the patient's surgical experience, reduce the cost of laparoscopic surgery, and reduce the occurrence of negative events in the operation.

## Methods

### Study design

The First Hospital of Qinhuangdao City began to upgrade the multimodal recognition technology in January 2020 to carry out innovative management of surgical departments. To investigate the effect of our upgraded multimodal identification technology in the innovative management operation department on laparoscopic surgery efficiency, surgical cost, and patient experience compared with our previous traditional management procedure, we conducted a cohort of laparoscopic surgeries. 2,280 cases of laparoscopic surgery using traditional surgical management procedures from January to December 2019 were set as the control group, and 2,350 laparoscopic surgeries from January to December 2020 with an information-based management process were selected as the experimental group. The sampling method of this study was cluster sampling. The control group was the traditional process group, and the experimental group was the multimodal practice group. The study was approved by the ethics committee of the First Hospital of Qinhuandao.

### Participant recruitment

Patient inclusion criteria: (1) hospitalized patients (2) patients who were transported to and from field staff (3) surgical patients requiring laparoscopic equipment. Participants were divided into traditional process groups and multimodal practice groups. The multimodal practice group is based on the entire operation process, and designs an intelligent information recognition system for the problems existing in weak links and key points, so that the information-based process can be compared with the traditional manual management mode for research. All participants have signed informed consent.

### Surgery management procedure

The multimodal surgical information recognition system is a part of the operating room information management. The information systems used in this study were already available and directly used. The multimodal surgical information recognition system establishes a data connection with the hospital management information system to create an intelligent management platform for the operating department and realize real-time information update. The specific content of the multi-modal surgical information recognition system includes staff card swiping, mobile phone application scanning code recognition, system-side appointments, reduced personnel operations, and reduced human interference factors (16).

Traditional group (control group): The traditional group uses traditional manual surgery management, including manual ordering before surgery, taking patients from the ward to the operating department, and then connecting to the operating room, manual arrangement of surgical laparoscopic equipment, and no nodes for the circulation and use of surgical equipment. After the patients were returned to the ward after surgery, the researchers collected data from multiple links and manually analyzed the relevant factors that restricted efficiency (17).

Multi-model group: Patients in the Multi-model group were managed by a multimodal surgical information recognition system. The multimodal surgical information recognition system adopts information technology for surgical management, and designs intelligent information processes, including task release process, task acceptance process, data statistics module, and function setting module (18). Before and after the operation, the nurses of the operating department use the mobile phone application to make an appointment to dispatch the order to the ward to pick up the patient. Field nurses start performing tasks by taking orders on the surgical line. The target time has been designed by the system prior to surgery, and the reasons for exceeding the time will be collected. When a patient is connected to the operating department, the order is deemed complete, and the computer in the background automatically aggregates the data. Laparoscopic devices are set up to share modules. Surgical instruments are positioned by scanning codes. Laparoscope usage times are accurately calculated and staggered. Data collection is carried out in multiple links, and the data is automatically analyzed in the background.

The time and human constraints of all setting links before, during, and after surgery were monitored, and data was collected and analyzed by the operation management department.

### Statistical analysis

Frequency or percentage was used to show the categorical variables. Mean and standard deviation were used for the presentation of continuous variables. Statistical analysis in this research was performed by student test and one-way ANOVA test with mean ± SD or SEM. *p* < 0.05 was considered as statistical difference.

## Results

### Demographics characteristics

The baseline data in the surgical department between 2,280 surgical patients in traditional procedure group and 2,350 surgical patients in multimodal practice group are presented in Table 1. As shown in Table 1, there is no statistical difference of the characteristics including gender, age, and primary diseases (*p* > 0.05).

**Table 1:** Demographic and clinical characteristics of patients under traditional process group and multimodal practice group.

Characteristics	Study group		*p*
	Tradition (*n* = 2,280)	Multi-model (*n* = 2,350)	
Gender			
Male	1,245 (54.6%)	1,247 (53.1%)	0.302
Female	1,035 (45.4%)	1,103 (46.9%)	
Age (years)	50.6 ± 15.2	51.9 ± 16.7	0.137
Primary disease			
Hepatobiliary surgery	673 (29.5%)	625 (26.6%)	0.082
Gynecological operation	248 (10.9%)	286 (12.2%)	
Gastrointestinal surgery	846 (37.1%)	911 (38.8%)	
Urologic surgery	485 (21.3%)	487 (20.7%)	
Others	28 (1.2%)	41 (1.7%)	

Values were expressed as *n* (percentage, %) or mean ± SD. *p* values for each group were derived from Mann–Whitney test. Chi-square test or Fisher's exact test was used for assessing distribution of observations or phenomena between two groups.

### Clinical characteristics of operation departments

The indexes in operation department under traditional process group and multimodal practice group are shown in Table 2. The number of first operations and consecutive operations, the number of sterile items, the number of packages of high-value consumables, the number of pieces of clothing consumed, the number of specimens submitted for inspection, and the number of bags of transfused blood products between the two groups were counted and analyzed.

**Table 2:** Clinical characteristics of operation department under traditional process group and multimodal practice group.

Characteristics	Study group	
	Tradition (*n* = 2,280)	Multi-model (*n* = 2,350)
Operation room (no.)	21	21
The first operation	251	248
The continuous operations	2,029	2,102
Sterile medical device (bag)	6,787	7,093
High-value consumable (unit)	905	895
Clothing consumption (unit)	2,737	2,518
Clinical samples (no.)	1,217	1,395
Blood product (bag)	551	496

### Operating efficiency of operating room

Evaluation of operating efficiency of operating room between traditional process group and multimodal practice group is shown in Table 3. Among the 251 cases in the control group, 198 cases were on time, and the rate of on-time operation was 78.9%; in the multimodal practice group, 229 cases were on time, and the rate of on-time operation was 92.3%. There was a significant difference in the rate of on-time operation between the two groups. The waiting times for consecutive surgeries were compared between the two groups. The multimodal practice group had significantly shorter waiting times for consecutive surgeries relative to the traditional group. In addition, the mean operative time for all procedures was significantly shorter in the multimodal practice group relative to the traditional group.

**Table 3:** Evaluation of operating efficiency of operating room between traditional process group and multimodal practice group.

Characteristics	Study group		*p*
	Tradition (*n* = 2,280)	Multi-model (*n* = 2,350)	
Punctually cut skin rate (the first operation)	198/251 (78.9%)	229/248 (92.3%)	<0.001
Waiting time (min) (the continuous operations)	58.6 ± 12.2	37.9 ± 13.7	<0.001
Operation time (min)	136.8 ± 31.2	112.5 ± 28.3	<0.001

Values were expressed as *n* (percentage, %) or mean ± SD. *p* values for each group were derived from Mann–Whitney test. Fisher's exact test was used for assessing distribution of observations or phenomena between two groups.

### Material management efficiency

Evaluation of material management efficiency between traditional process group and multimodal practice group is presented in Table 4. There is a statistical difference between the two groups in the billing omission rate of high-value consumables. The multimodal practice group also significantly improved the quality tracking of sterile items used in surgery, such as instruments, consumables, etc. The wear of clothing accessories and other materials was significantly reduced in the multimodal practice group.

**Table 4:** Evaluation of material management efficiency between traditional process group and multimodal practice group.

Characteristics	Study group		*p*
	Tradition (*n* = 2,280)	Multi-model (*n* = 2,350)	
Missing accounts of high value consumables	8 (0.35%)	0 (0%)	0.003
Tracking of sterile items	1,847 (81.0%)	2,248 (95.7%)	<0.001
Detrition of clothing	286/2,737 (10.4%)	124/2,518 (4.9%)	<0.001

Values were expressed as *n* (percentage, %). *p* values for each group were derived from Fisher's exact test.

### Comparison of waiting time for blood product injection and submission time of clinical samples

**Figure 1:**
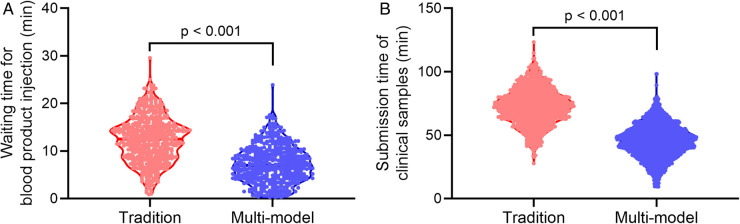
Comparison of waiting time for blood product injection (**A**) and submission time of clinical samples (**B**) between traditional process group (tradition) and multimodal practice group (multi-model). Violin plot. Mann–Whitney test.

The time of blood product verification and specimen submission time of the two groups of surgical patients was compared between the two groups. As shown in Figure 1, the multimodal practice group was able to significantly reduce blood product verification time and specimen delivery time compared with the traditional group.

### Patients’ satisfaction

Comparison of patients’ satisfaction between traditional process group and multimodal practice group was shown in Table 5. The patient experience and satisfaction were surveyed by the operating room nurses who performed the preoperative visit during the postoperative return visit (within 3 days after the operation), and the patients were asked about their satisfaction with the whole operation from admission to the operation and the nursing process after the operation. The multimodal practice group was able to significantly improve patient satisfaction.

**Table 5:** Comparison of patients’ satisfaction between traditional process group and multimodal practice group.

Characteristics	Study group		*p*
	Tradition (*n* = 2,280)	Multi-model (*n* = 2,350)	
Very satisfied	708 (31.1%)	974 (41.5%)	<0.001
Satisfied	964 (42.3%)	893 (38%)	
Basically satisfied	477 (20.9%)	386 (16.4%)	
Not satisfied	131 (5.7%)	97 (4.1%)	

Values were expressed as *n* (percentage, %). *p* values for each group were derived from Chi-square test.

## Discussion

The optimization of surgical procedures and the management of surgical quality and safety have been the focus of current multinational hospital managers (19–21). Patient information and surgical consumables can be connected to the hospital's Internet platform for information exchange and communication, through radio frequency identification (RFID), two-dimensional codes, infrared sensors, laser scanners, mobile personal digital assistants and other information sensing devices (22, 23). The big data-based surgical management method can realize the intelligent identification, positioning, tracking, monitoring and management of single surgical equipment (24). The multimodal surgical information identification system integrates and interconnects the multi-source data and information island systems of various departments in the hospital, and builds the quality and safety information management system of the hospital operation department together with the management process of the operation department (25).

Our hospital has launched multimodal recognition technology to carry out innovative management of the surgical department since 2019. Our multimodal surgical information recognition system is a part of the operating room information management. The multimodal surgical information recognition system establishes a data connection with the hospital management information system to create an intelligent management platform for the operating department and realize real-time information update (26). The specific content of the multi-modal surgical information recognition system includes staff card swiping, mobile phone application scanning code recognition, system-side appointments, reduced personnel operations, and reduced human interference factors (27).

We believe that our multi-model identification system has important implications for improving the efficiency of clinical work. We hope to realize the evaluation of information accuracy through the application of the multimodal surgical information recognition system, thereby greatly reducing the time and labor cost. For instance, the quality of the preparation of laparoscopic surgery-related instruments directly affects the operation and the effect of the operation. Therefore, laparoscopic surgery should strengthen the management of the preparation of laparoscopic surgical instruments. In this study, we investigate the effect of application of multimodal identification technology in the innovative management operation department on laparoscopic surgery efficiency, surgical cost, and patient experience. Patients in our hospital were currently managed by a multimodal surgical information recognition system. The multimodal surgical information recognition system adopts information technology for surgical management, and designs intelligent information processes, including task release process, task acceptance process, data statistics module, and function setting module. Before and after the operation, the nurses of the operating department use the mobile phone application to make an appointment to dispatch the order to the ward to pick up the patient. Field nurses start performing tasks by taking orders on the surgical line. The target time has been designed by the system prior to surgery, and the reasons for exceeding the time will be collected. When a patient is connected to the operating department, the order is deemed complete, and the computer in the background automatically aggregates the data. Laparoscopic devices are set up to share modules. Surgical instruments are positioned by scanning codes. Laparoscope usage times are accurately calculated and staggered.

## Conclusion

In conclusion, we demonstrate that application of multimodal identification technology in the innovative management operation department on laparoscopic surgery efficiency, surgical cost, and patient experience. Our findings show that the newly implemented multimodal surgical information recognition system in our hospital can significantly improve the operating efficiency of laparoscopic operating room compared to traditional surgery and nursing procedures. The multimodal surgical information recognition system can also significantly improve material management efficiency and reduce the wastage of surgical instruments in the laparoscopic operating room. We also demonstrated that the multimodal practice group was able to significantly reduce blood product verification time and specimen delivery time in surgical patients. In addition, patients who were managed by the multimodal surgical information recognition system had significantly lower waiting time for surgery, and their surgical experience was significantly improved. We hope that our study can provide new research evidence for the generalized use of multimodal surgical information recognition systems.

## Data Availability

The raw data supporting the conclusions of this article will be made available by the authors, without undue reservation.
